# Visuomotor interactions in the mouse forebrain mediated by extrastriate cortico-cortical pathways

**DOI:** 10.3389/fnana.2023.1188808

**Published:** 2023-05-09

**Authors:** Karoline Hovde, Ida V. Rautio, Andrea M. Hegstad, Menno P. Witter, Jonathan R. Whitlock

**Affiliations:** ^1^Kavli Institute for Systems Neuroscience, Norwegian University of Science and Technology, Trondheim, Norway; ^2^Faculty of Health and Medical Sciences, University of Copenhagen, Copenhagen, Denmark

**Keywords:** mouse, anterograde tracer injections, retrograde AAV injections, immunohistochemistry, visual cortex, extrastriate, motor cortex

## Abstract

**Introduction:**

The mammalian visual system can be broadly divided into two functional processing pathways: a dorsal stream supporting visually and spatially guided actions, and a ventral stream enabling object recognition. In rodents, the majority of visual signaling in the dorsal stream is transmitted to frontal motor cortices via extrastriate visual areas surrounding V1, but exactly where and to what extent V1 feeds into motor-projecting visual regions is not well known.

**Methods:**

We employed a dual labeling strategy in male and female mice in which efferent projections from V1 were labeled anterogradely, and motor-projecting neurons in higher visual areas were labeled with retrogradely traveling adeno-associated virus (rAAV-retro) injected in M2. We characterized the labeling in both flattened and coronal sections of dorsal cortex and made high-resolution 3D reconstructions to count putative synaptic contacts in different extrastriate areas.

**Results:**

The most pronounced colocalization V1 output and M2 input occurred in extrastriate areas AM, PM, RL and AL. Neurons in both superficial and deep layers in each project to M2, but high resolution volumetric reconstructions indicated that the majority of putative synaptic contacts from V1 onto M2-projecting neurons occurred in layer 2/3.

**Discussion:**

These findings support the existence of a dorsal processing stream in the mouse visual system, where visual signals reach motor cortex largely via feedforward projections in anteriorly and medially located extrastriate areas.

## Introduction

A common feature of visual cortical organization across mammals is that visual signals from the eye enter primary visual cortex (V1) via the thalamus, then travel to higher visual areas (Frost and Caviness, 1980; Benevento and Standage, 1982; Simmons et al., 1982; Kaas and Krubitzer, 1991; Salin and Bullier, 1995; Rosa and Krubitzer, 1999) which process progressively distinct components of visual stimuli (Maunsell and Newsome, 1987; Nassi and Callaway, 2009; Niell, 2015; Froudarakis et al., 2019). In primates and carnivores, higher visual processing streams collect into functionally divergent “dorsal” and “ventral” pathways, with the former supporting spatial and visually guided motor behaviors, and the latter enabling object recognition (Goodale and Milner, 1992; Nassi and Callaway, 2009). Parallel studies in rats, mice and hamsters have also uncovered anatomical (Olavarria et al., 1982; Olavarria and Montero, 1989; Montero, 1993; Wang and Burkhalter, 2007; Wang et al., 2012; Zingg et al., 2014), physiological (Montero and Jian, 1995; Andermann et al., 2011; Marshel et al., 2011; Glickfeld et al., 2013; Garrett et al., 2014; Han et al., 2022) and behavioral (Schneider, 1969; Mlinar and Goodale, 1984; Kolb and Walkey, 1987; Save et al., 1992; Tees, 1999; Ho et al., 2011) evidence supporting a dorsal-*versus*-ventral organization in the rodent visual system, though with fewer functionally specialized nodes. The notion of a dorsal stream in rodents has been further supported by work demonstrating causal contributions of higher visual cortical projections to fine-grained visuomotor control in midline motor cortex in mice (Itokazu et al., 2018), and has prompted investigations into the role of dorsal stream pathways in the production and perception of naturalistic actions (Tombaz et al., 2020; Viaro et al., 2021) and spatial navigation (McNaughton et al., 1989).

Although in mice it has been established that several of the ∼10 (Wang and Burkhalter, 2007; Froudarakis et al., 2019) higher-visual, or extrastriate, areas send direct projections to frontal and midline motor cortices (Wang et al., 2012; Zingg et al., 2014; Itokazu et al., 2018), several pieces of the puzzle remain missing regarding the anatomical chain by which cortical signaling propagates from V1 to motor areas. For example, it is not known whether the output from V1 is uniformly distributed across frontally-projecting extrastriate cortex, if there are regional preferences among these areas or if there is a laminar profile characteristic of such projections.

We sought to address these questions here using a dual pathway tracing approach in which efferent fibers from V1 were labeled using the anterograde tracer 10 KD biotinylated dextran amine (BDA), and motor-projecting extrastriate neurons were labeled via retrogradely traveling, recombinant AAV2/1-retro (rAAV-retro) (Tervo et al., 2016) in secondary motor cortex (M2). Viral injections were targeted to the posterior sector of M2 which receives visual input (Reep et al., 1990; Zingg et al., 2014) and controls movement of the eyes, head and vibrissae (Donoghue and Wise, 1982; Sinnamon and Galer, 1984; Brecht et al., 2004). We visualized the areal overlap of retrograde and anterograde labeling in whole-hemisphere flattened sections of cortex, which revealed a characteristic arrowhead shape of M2-projecting neurons along the anterior perimeter of V1. M2 projecting neurons co-occurred with V1 output fibers in the anteromedial (AM), posteromedial (PM), rostrolateral (RL), and anterolateral (AL) extrastriate areas. In coronal sections, retrograde labeling from M2 spanned superficial and deep layers in anterior extrastriate areas, appearing pillar-like, but became progressively more superficial at farther posterior locations. Morphological 3D reconstructions revealed substantial putative connectivity between V1 axons and M2-projecting neurons in both superficial and deep layers, but with a markedly higher incidence in layer 2/3. Together, our results show that V1 output bound for motor cortex is broadcast non-uniformly across extrastriate regions and is relayed via abundant feedforward projections, particularly in layer 2/3.

## Materials and methods

Eight female C57BL/6JBomTac mice (23–25 g, Taconic) and one male C57BL/6JBomTac mouse (33 g) were used in the project. Five animals received injections of virus and tracer into the left hemisphere and their brains were cut in tangential flattened sections. One brain was excluded from the analysis due to poor uptake of the tracer. Four animals received similar injections in the right hemisphere and the brains were cut in coronal sections. One brain was excluded due to a misplaced injection in V1. All animals were housed in single cages, kept on a reversed day-night cycle, and given *ad libitum* access to food and water. The surgical procedures were approved by the Norwegian Food Safety Authority and the local Animal Welfare Committee of the Norwegian University of Science and Technology and followed the European Communities Council Directive and the Norwegian Animal Welfare Act.

### Retrograde viral tracing and anterograde anatomical tracing

For stereotaxic surgeries, the initial coordinates for V1 and M2 injections were calculated in accordance with Paxinos and Franklin (2012) and adjusted based on previous injections in-house. The animals were deeply anesthetized with isoflurane throughout the surgery and their body temperature was kept stable at 37°C. Local anesthetic Marcain (1–3 mg/kg, bupivacaine, AstraZeneca) was injected above the skull, and analgesics Temgesic (0.1 mg/kg, buprenorphine, Indivior, Chesterfield, VA, USA) and Metacam (5 mg/kg, meloxicam, Boehringer Ingelheim, Vetmedica, Germany) were given subcutaneously. After shaving and disinfecting the head (70% ethanol; iodine, NAF Liniment 2%, Norges Apotekerforening), an incision was made along the midline and the skull was cleaned (hydrogen peroxide, H_2_O_2_; 3%, Norges Apotekerforening), the height of bregma and lambda were measured and adjusted along the anterior-posterior axis to ensure the skull was leveled and two craniotomies were made at the coordinates for injections into secondary motor cortex (M2; AP: + 0.3, ML: + 0.5, DV: −0.5) and primary visual cortex (V1; AP: −4.5, ML: + 2.3, DV: −0.30–0.60) in either the left (*N* = 5) or right (*N* = 4) hemisphere. A retrograde GFP-tagged adeno-associated virus rAAV2/1-retro (retrograde AAV-CAG-GFP; serotype “retro,” Addgene, Cat. # 37825) was pressure injected into M2 (170, 180, 250, and 400 nL volume injections) by use of glass capillaries [World Precision Instruments (WPI), Cat. No. 4878] and Micro4 pump (WPI; speed 35 μL/s), and the capillary was kept in place for 10 min after the injection, to minimize leakage of the virus. An anterograde tracer, 10 KD biotinylated dextran amine [BDA, Dextran, Biotin, 10,000 MW, Lysine Fixable (BDA-10,000), Thermo Fisher Scientific Cat. No. D1956, RRID:AB_2307337 in 5% solution in 0.125 M phosphate buffer], was injected into V1 iontophoretically by pulses of positive DC-current (6 s on/off alterations, 6 μA, 10 min) using glass micropipettes (20 μm tip, Harvard apparatus, 30-0044). After the injection was completed, the craniotomies were filled with Venus Diamond Flow (Kulzer, Mitsui chemical group, Cat. # 879566), the skull was cleaned and the skin was sutured and disinfected with iodine. The animal was kept in a heated chamber until awake and active. Post-operative analgesic (Metacam; 5 mg/kg) was given 12 h post-surgery and the health of the animal was closely monitored the days after surgery.

### Perfusion and tissue processing

All animals were killed and perfused 21 days post-surgeries.

#### Tangential flattened sections

The animals that received injections in the left hemisphere were given an overdose of pentobarbital (0.2 mL/100 g) and transcardially perfused using fresh ringer’s solution (0.025% KCl, 0.85% NaCl, 0.02% NaHCO_3_, pH 6.9) and PFA (1%, 0.125 M phosphate buffer, pH 7.4), and the brains were carefully removed and kept in a cup of PFA. Within 1 h, the cortex of the left hemisphere was dissected out and flattened, and tangential sections (50 μm) were prepared. To do so, the intact brain was cut along the midline, subcortical areas and cerebellum were removed, and one cut was made in the fornix dorsal to the anterior commissure. Horizontal cuts were then made along the white matter, and relief cuts were made ventral to postrhinal cortex and in the anterior cingulate cortex. The hippocampus was unfolded, and the cortex was flattened between two microscope glasses covered with parafilm (Laboratory film, Pechiney, Plastic packaging, Chicago, IL, USA) and submerged in PFA (4%) overnight at 4°C with a glass weight on top (52 g). The following day, the flattened cortex was removed from the microscope slides and left in a cryoprotective dimethyl sulfoxide solution (2% dimethyl sulfoxide, DMSO, in 0.125 M phosphate buffer; VWR) overnight. The flattened cortex was then cut in 50 μm tangential sections in one series on a freezing microtome (Microm HM430, Thermo Scientific, Waltham, MA, USA).

#### Coronal sections

Following the same procedure as above, animals with right hemisphere injections were perfused with fresh ringer’s solution and PFA (4%). The brain was placed in a container with PFA (4%) overnight, transferred to cryoprotective solution (DMSO, 2%) and stored overnight. The brain was cut on a freezing microtome in 40 μm sections in three series. The first series was mounted on Superfrost Plus microscope slides (Gerhard Menzel GmbH, Braunschweig, Germany) and used for Nissl staining, the second was processed to reveal the tracer and virus, and the third was stained with 3.3′-Diaminobenzidine tetrahydrochloride (DAB, Sigma-Aldrich, St. Louis, USA) against the muscarinic acetyl choline receptor 2 (M2AChR2) or kept as a backup in cryoprotective solution stored at −24°C.

### Histology and immunohistochemistry

#### Nissl

Series one of the coronal sections was stained with Nissl staining. To do so, sections were hydrated in running water and dehydrated in baths with increasing percentage of ethanol (50, 70, 80, 90, and 100% x3), cleared in a solution of xylene (2 min; VWR, International, Fontenay-sous-Bois, France) and rehydrated in decreasing percentage of ethanol, followed by a brief rinse in running water prior to staining in Cresyl violet on a shaker (3 min). The sections were rinsed in water, differentiated in a solution of ethanol/acetic acid (0.5% acetic acid in 70% ethanol; VWR, International, Fontenay-sous-Bois, France) until reaching the desired staining contrast, and cleared in two xylene baths (2 min, 20 min) before being coverslipped with an entellan-xylene solution (Merck KGaA, Darmstadr, Germany).

#### BDA visualization and enhancement of rAAV-retro signal

All flattened tangential sections and series two of the coronal sections were processed to reveal the BDA tracer and to enhance signal from the virus using a 2-day immunohistochemical procedure. On day one, the sections were washed in phosphate buffered saline (PBS; 3 × 5 min), followed by a phosphate buffered saline solution with Triton (PBS 0.1 M, 0.3% Triton, 3% BSA; 2 × 10 min) on a shaker (100 rpm) at room temperature (RT). The sections were incubated with anti-GFP primary antibody (GFP; rabbit anti-GFP, 1:1,000, Thermo Fisher Scientific, A-11122) overnight on a shaker (60 rpm) at 4°C. On day two, the sections were washed in PBS solution (PBS 0.1 M, 0.3% Triton, 3% BSA; 2 × 5 min) and incubated with secondary antibody (Alexa Fluor 488-tagged goat anti-rabbit Ab, 1:1,000, Thermo Fisher Scientific, A-11008) and with Alexa Fluor 633-conjugated Streptavidin (1:400, Thermo Fisher Scientific Cat. No. S-21375, RRID:AB_2313500) against BDA on a shaker (60 rpm) at RT (75 min). The sections were rinsed in Tris buffer 0.606% [Tris(hydroxymethyl)aminomethane, pH 7.6; 3 × 10 min], mounted on non-frost microscope slides using a Tris-gelatin solution (0.2% gelatin in Tris-buffer, pH 7.6) and coverslipped with an entellan-xylene solution.

#### DAB staining against M2AChR

Series three of the coronal sections were stained with 3.3′-Diaminobenzidine tetrahydrochloride (DAB, Sigma-Aldrich, St. Louis, MO, USA) to visualize M2AChR density and were used only for delineation purposes. To do so, sections were rinsed in PBS (0.125 M, 2 × 5 min) followed by TBS-Tx (2 × 5 min) and incubated with primary antibody (Rat anti-muscarinic M2 monoclonal antibody, unconjugated, clone m2-2-b3, 1:750, Millipore Cat. No. MAB367, RRID:AB_94952; overnight at RT), washed in TBS-Tx (2 × 5 min) and incubated with mouse-absorbed, rabbit-anti-rat secondary antibody [Anti-rat IgG (H + L), 1:300, Vector Laboratories Cat. No. BA-4001, RRID:AB_10015300] for 90 min at RT. The sections were washed in TBS-Tx (2 × 5 min), in PB (2 × 5 min), in H_2_O_2_-metanol solution (0.08%, Sigma-Aldrich, 2 × 5 min) and in TBS-Tx (2 × 5 min) and incubated with a Vector ABC kit (Vector laboratories, Inc., Burlingame, CA, USA) for 90 min at RT, per the manufacturer’s instructions. They were then washed in TBS-Tx (2 × 5 min) and Tris-buffer (2 × 5 min) before being incubated with DAB (10 mg in 15 mL Tris- buffer, Sigma-Aldrich) at RT. H_2_O_2_ (2 μL, 30%, Sigma-Aldrich) was added to the DAB solution immediately prior to the incubation. The solution was filtered, the sections were incubated in DAB until the desired level of staining was reached and washed in Tris-buffer solution. A 0.2% gelatin solution was used to mount the sections on Menzel glass slides, the slides were dried overnight on a heated pad and coverslipped with an entellan-xylene solution.

### Imaging and analyses

All Nissl and M2AChR stained sections were digitized for analyses using a bright field scanner (Zeiss Axio Scan.Z1). Sections with fluorescence labeling were examined in a fluorescence microscope (Zeiss Axiomager M2) and digitized with a fluorescence scanner (Zeiss Axio Scan.Z1). Lower exposure time was used for the sections with the injection sites in V1 and M2 to avoid saturation of the signal.

High resolution images (63× oil) in z-stacks (typically 70–90 planes, 0.14 μm intervals, 0.05 μm pixel size) were taken of selected sections with fluorescence labeling using a Zeiss confocal microscope (LSM800). The images we deconvoluted in Huygens 19.10 (Scientific Volume Imaging) using the default express deconvolution. The deconvoluted image stack was saved as 16 bit.pic files (one for each fluorescent channel) and opened in Neurolucida360 (MBF Bioscience) for reconstruction.

**FIGURE 1:**
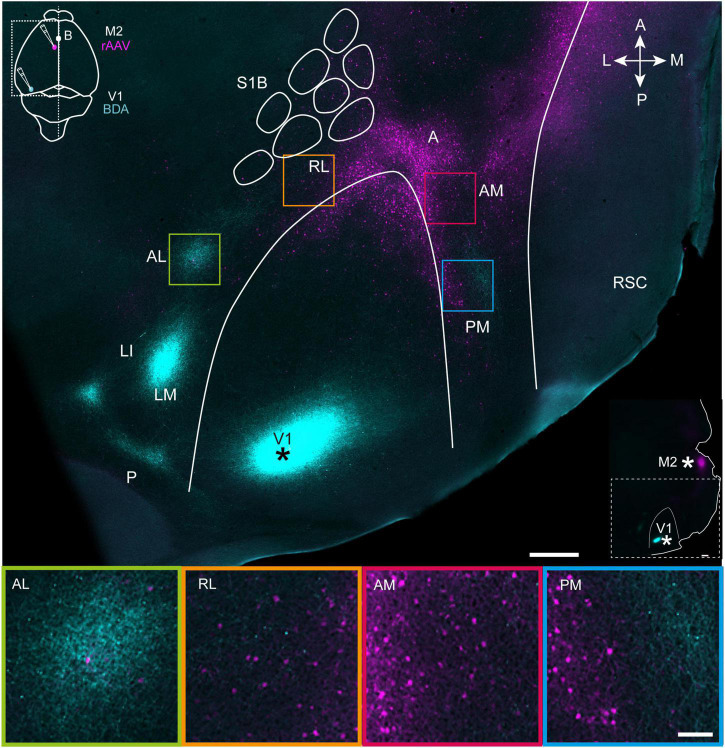
Projections from V1 (cyan) and M2-projecting neurons (magenta) viewed in a representative tangential section through superficial layers in flattened dorsal cortex. **Top**, a tangential section through layer 2/3 shows the BDA injection site in V1 (marked with asterisk; see also inset at top left) and projections to extrastriate areas at the periphery of V1. Projection neurons targeting M2 are shown in magenta (injection schematic shown in inset), and colocalized with V1 efferent fibers in areas AL, RL, AM, and PM. The outlines of V1 and the S1 barrel fields were traced using myeloarchitectonic patterns and M2AChR staining from a neighboring section in layer 4 (Methods). Note, a shorter exposure time was applied when scanning the injection site than for projections to avoid signal saturation (Methods). **Bottom**, magnification of extrastriate areas highlighted in the flattened section above. See list for abbreviations. The inset at right indicates injection sites in V1 and M2 with asterisks. White scale bar in upper image = 500 μm; in lower image = 100 μm.

The outlines of V1 and S1B were drawn on flattened tangential sections using myeloarchitectonic features visible in layer IV (Supplementary Figure 1). The same outlines were copied and overlaid on sections cut through superficial layers of cortex (Figure 1), where myeloarchitectonic features were not present.

### Reconstruction and proximity testing

The deconvoluted image stacks obtained from Huygens 19.10 (see above) were opened and the two fluorescence channels were merged in Neurolucida360. The black point of the image was increased 10%, the white point lowered 90% and gamma was set to 1.20 for visualization purposes, as this enhanced image contrast and removed background noise. Dendrites were traced using the “user-guided tracing” mode with the method “voxel scooping.” Specifically, the user traced the dendrites in the image manually with a computer mouse to identify which parts of the image the software would reconstruct, after which spines were detected automatically using the nearest branch mode. The specifications for detecting spines were: outer range = 0.5, Detector sensitivity = 90%, Minimum count = 50, Minimum height = 0.3. The collections of automatically detected spines were subsequently inspected and manually curated. Next, axons were traced manually using the tracing option “direction kernels” and boutons were detected automatically using the nearest branch mode. The typical process width for these methods was 0.77 μm. After the reconstruction was complete, synaptic markers were placed using the “synaptic markers” button with a 0.25 μm requirement and the results were saved as a.DAT file. The file was opened in Neurolucida explorer and a branch structure analysis was performed using the synapses mode and synaptic markers details. The markers with a distance below 0.25 μm were considered as putative synaptic contacts and used in subsequent comparisons between areas. The soma was not reconstructed in Neurolucida but imported as a 2D image into the final 3D reconstruction of neurons for illustration purposes only.

## Results

### Areal organization of V1 output and M2 input

First, we sought to gain an overview of cortical regions where efferent fibers from V1 and cell bodies of motor-projecting extrastriate neurons were colocalized. To do so, four mice received unilateral injections of BDA targeted to the posterior pole of V1 (Figure 1, top panel), which previous work has shown sends projections to all downstream visual areas (Olavarria and Montero, 1989; Wang and Burkhalter, 2007). The same animals received rAAV-retro injections in the posterior sector of secondary motor cortex, M2 [per the nomenclature of Paxinos and Franklin (2012)], due to the high density of visual input it receives in mice and rats (Reep et al., 1990; Wang et al., 2012). 3 weeks after surgery, the brains were removed and the left hemisphere was dissected out, flattened and cut tangentially into sections parallel to the brain surface, allowing us to visualize regional labeling of efferent V1 fibers and M2-projecting neurons in extrastriate cortices. At least seven extrastriate areas were discernable based on the topographical positioning and orientation of projection plexuses relative to V1, with the most prominent labeling from V1 in LM, LI, AL, and PM, with more moderate labeling in RL and AM, and the weakest labeling in area A [Figure 1, Top; regional nomenclature per (Olavarria et al., 1982; Wang and Burkhalter, 2007)]. The location of V1 projections to extrastriate areas was consistent across mice, though the relative strength of labeling within regions varied depending on the mediolateral location of the injections in V1 (Supplementary Figure 1) and was therefore not quantified here, but can be found in earlier work (Wang and Burkhalter, 2007). The weak labeling in area A could also have been due the injection locations in V1, so it was not analyzed further, despite being an established component of the mouse dorsal visual stream (Wang et al., 2012).

Retrogradely labeled M2-projecting neurons were condensed at the anterior pole of V1, and flanked its medial and lateral borders in a V-shape that in some cases continued as far laterally as area AL, and as far posteriorly as area PM (Figure 1, top and Supplementary Figures 1, 2). Regions showing the most extensive coincident labeling from V1 and to M2 were AM, PM and RL, which partly overlapped with posterior parietal cortex (PPC) (Hovde et al., 2018; Gilissen et al., 2021), and sparse labeling of M2-projecting neurons was present area AL. In all extrastriate regions, dual labeling of V1 efferent fibers and M2-projecting neurons was strongest in superficial layers and layer 4, with sparser labeling in deeper layers (Supplementary Figure 2), and this pattern was investigated in more detail in coronal sections.

### Laminar organization of V1 output fibers and M2-projecting neurons

**FIGURE 2:**
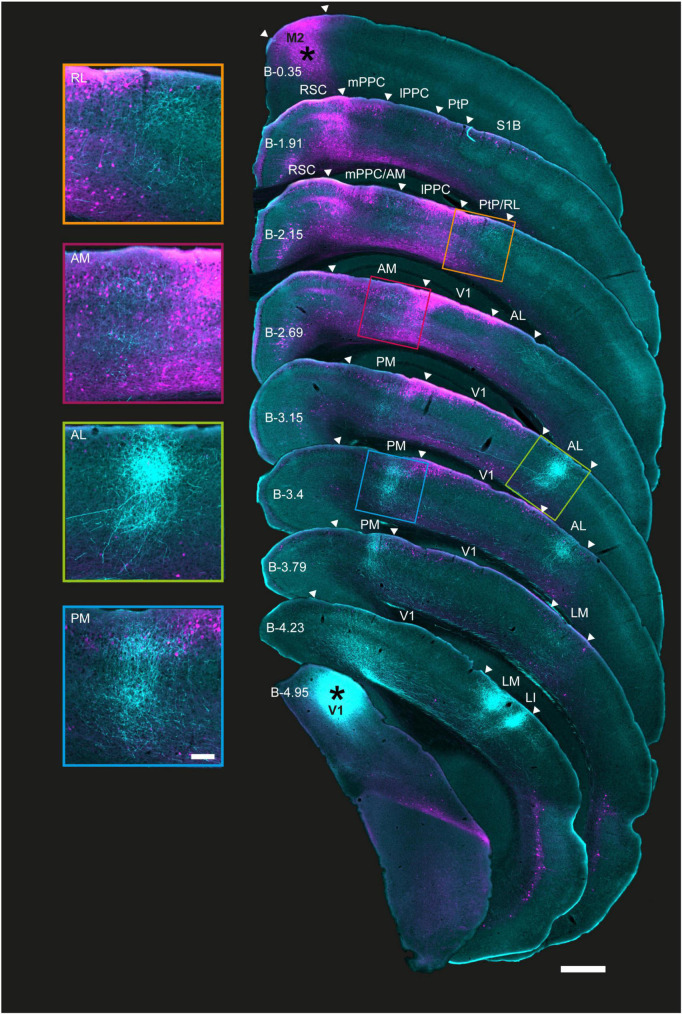
Coronal sections showing the laminar profile of BDA-labeled V1 projections and rAAV-retro-labeled M2-projecting neurons in posterior cortices. **Right**, low magnification series of coronal sections from the right hemisphere arranged from anterior (top) to posterior (bottom); injection sites marked with asterisks. Extrastriate areas and PPC boundaries are indicated by white triangles; PPC, its sub-areas, and V1 were delineated using adjacent Nissl and immunohistochemically stained sections from the same series. Anterograde and retrograde labeling from V1 and M2 colocalized in areas RL, AM, and PM, including in PPC, as well as area AL. M2 projecting neurons were found in superficial layers in all regions in the series, and extended to deep layers at more anterior locations, especially in RL and AM. **Left**, magnified view of extrastriate areas highlighted in sections to the right. As with Figure 1, shorter exposure times were used for injection sites than projections to avoid signal saturation (Methods); the figure is for illustration purposes. Approximate bregma coordinates (B; Paxinos and Franklin, 2012) are noted on each section; scale bar = 500 μm.

Similar injections of BDA and rAAV-retro were made in the right hemisphere of V1 and M2 in four additional mice, and coronal sections were collected in cortical regions spanning anteriorly from M2 to the posterior extent of V1 (Figure 2, right and Supplementary Figures 3, 4). Consistent with our observations in flattened sections, regions with the densest axonal plexuses from V1 were LM, LI, AL, and PM, with more moderate but clear labeling in AM and RL (Figure 2, left and Supplementary Figures 3, 4). V1 projections were observed in both superficial and deep layers of all extrastriate areas, though across animals we noted axonal fibers were concentrated in layers 3 and 5 (Figure 2, left, magnifications and Supplementary Figures 3, 4). As with flattened sections, there were very few axonal fibers from V1 in area A.

Across animals, rAAV-retro labeling from M2 was abundant in several extrastriate regions and spanned in a pillar-like fashion from layers 1 to 6 in more anterior regions. At its anterior extent, retrograde M2 labeling formed an apparent pillar at the border of the medial PPC (mPPC) and agranular retrosplenial cortex (RSC; Figure 2, 2nd coronal section), then, progressing posteriorly, split into medial and lateral branches that overlapped with mPPC and AM medially, and lateral PPC (lPPC) and RL laterally. Retrograde labeling from M2 was strong in superficial and deep layers in all PPC sub-regions as well as PM and AL as far posterior as Bregma (B) level −3.15 [Figure 2 and Supplementary Figures 3, 4, 4th, and 5th coronal sections; Bregma location based on Paxinos and Franklin (2012)]. Further posteriorly, strong retrograde labeling persisted mostly in superficial layers 1–3 (≥B −3.15) in PM. Laterally, a similar pattern was observed in AL and V1, with the exception that layer 5 was almost devoid of neuronal labeling and layer 6 showed only sparse neuronal labeling (Figure 2, 5 and 6th sections; magnified insets, left). Retrograde labeling tapered off completely in all layers at the farthest posterior locations, with no M2-projecting neurons remaining in areas LM or LI, or at the posterior extent of V1 (≥B −4.23; Figure 2, right). Thus, of the six identifiable extrastriate areas in this tissue series, regions RL, AM, PM, and AL, in addition to the anterior-most portion of V1, were potential nodes at which visual signals were conveyed to secondary motor cortex.

### Neuronal reconstructions and localization of putative synaptic contacts

**FIGURE 3:**
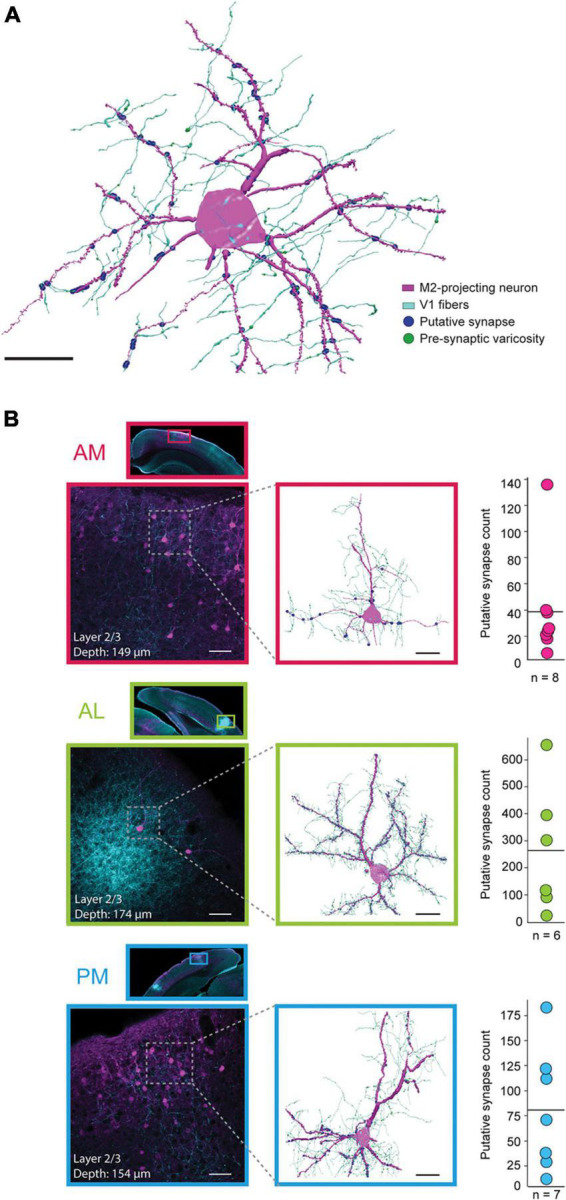
Anatomical reconstructions and proximity analysis of V1 axons and M2-projecting neurons in extrastriate areas AM, AL, and PM. **(A)** Representative reconstruction of a single M2-projecing pyramidal neuron from area AL (magenta) receiving synaptic input from V1 axons (cyan); putative synaptic contacts are shown as blue circles and pre-synaptic varicosities are visualized as green enlargements on the V1 axons (varicosities are not visible at points of putative contact due to overlapping blue circles). Black scale bar = 20 μm. **(B)** Top row, inset, a low magnification scan of a coronal tissue section highlighting the portion of area AM where the reconstruction was performed. Top row, lower left, a higher magnification field of view of area AM shown above. Top row, middle, reconstruction of the neuron in the stippled box to the left, with the same labeling convention as the example in panel **(A)**. The depth (from cortical surface to soma center) and cortical layer of each neuron are included in the inset for each example. Top row, right, mean (solid line) and distribution of putative synapses for all reconstructed neurons in area AM. Middle row, same as top, but for as area AL. Bottom row, same as upper rows, but for area PM. Black scale bar = 20 μm. White scale bars = 50 μm.

The colocalization of V1 fibers and M2-projecting neurons in specific extrastriate areas seen here, along with observations from previous studies (Wang et al., 2012; Zingg et al., 2014), suggest these regions serve as nodes for the propagation of visual information to the motor system. However, overlap *per se* does not mean that synaptic connections are in fact present, so we tested this possibility more directly. To do so, we stacked high-magnification (63×) serial confocal scans and created 3-dimensional morphological reconstructions in all extrastriate areas in which M2-projecting neurons had clearly evident pre-synaptic V1 fibers in their vicinity (Figure 3A; Methods). Because V1 neurons were labeled by extracellular tracer injections, it is likely that fibers from multiple V1 neurons contributed to each reconstruction. We also note that the resulting reconstructed cells were biased in that they were chosen from areas that had colocalized labeling, which here were AM, AL, PM, and RL, and we do not exclude that similar overlap could occur in area A. Neurons were selected for reconstruction based on three criteria: (i) the presence of a clear and completely filled neuronal soma associated with (ii) filled, long spiny dendrites, and (iii) a sufficiently densely labeled axonal plexus from V1 overlapping the dendrites, where connectivity was expected to occur (see examples in magnified insets in Figure 3B). In all sub-areas, neurons meeting these criteria spanned cortical depths ranging from 90 to 640 μm but, due to the preponderance of retrograde labeling from M2 in superficial layers, the majority of reconstructed cells came from layer 2/3. Once sufficiently-labeled cells were identified, putative synapses were identified using close spatial proximity (<0.25 μm) between axonal varicosities and dendritic spines as a proxy for synaptic contacts (Methods) (Wouterlood et al., 2008; Koganezawa et al., 2015), with group data generated for area AM (*n* = 8 neurons from 3 mice), AL (6 neurons, 2 mice), and PM (7 neurons, 3 mice). Only one mouse had coincident anterograde and retrograde labeling in area RL, so it was excluded from the reconstruction analysis.

Area AM, which had high density retrograde labeling from M2, had the lowest mean number of putative V1 synapses per neuron (Figure 3B, top; 38.8 ± 13.7 (mean ± SEM); median = 25; range of 7 to 136 contacts per neuron; all reconstructed AM neurons shown in Supplementary Figure 5). Area AL, on the other hand, typically contained few M2-projecting neurons, but had the highest rate of V1 putative contacts of the 3 regions (Figure 3B, middle; mean = 263.8 ± 96.8; median = 209.5; range of 23 to 655 per neuron; all reconstructions shown in Supplementary Figure 6). In PM, the number of putative synaptic connections from V1 were intermediate between AM and AL across animals (Figure 3B, bottom; mean = 81 ± 28.6; median = 71; range of 12 to 183; all neurons shown in Supplementary Figure 7). As noted above, the large majority of neurons were reconstructed from superficial layers (*n* = 6 of 8 neurons in AM; 5 of 6 in AL; 6 of 7 in PM), due to sparse retrograde labeling and very few incidents of nearby V1 axonal processes in layers 5 and 6 (Supplementary Figures 5–7).

## Discussion

We used a dual anterograde and retrograde labeling strategy to characterize the regional intersection and connectivity patterns of V1 output fibers onto motor cortical-projecting neurons in mouse extrastriate cortex. We prepared flattened sections, which provided an overview of the entire dorsal cortex, and found that visual and motor pathways overlapped specifically in extrastriate and posterior parietal regions which support visuospatial and motor behavior in rodents (Schneider, 1969; Kolb and Walkey, 1987; McNaughton et al., 1989; Save et al., 1992; Chen et al., 1994; Kolb et al., 1994; Tees, 1999; Nitz, 2006; Harvey et al., 2012; Whitlock et al., 2012; Wilber et al., 2014; Yang et al., 2017; Itokazu et al., 2018). These findings confirm and extend observations in separate studies characterizing efferent projections from V1 (Olavarria et al., 1982; Wang and Burkhalter, 2007) and anterograde projections from extrastriate areas to motor cortices (Wang et al., 2012; Zingg et al., 2014) by directly demonstrating the physical overlap between visual and motor pathways. We additionally generated coronal sections, which revealed pillar-like labeling of M2-projecting neurons from superficial to deep layers in anterior AM and RL, which receded into mainly superficial layers posteriorly in PM, AL and in V1. Higher resolution analyses showed that retrogradely-labeled M2-projecting neurons were more frequent in layer 2/3 than layer 5 and reconstructions of putative synaptic connections showed a trend for higher rates of connectivity in superficial than deep layers.

Though our observations were consistent across animals, there were methodological limitations in the study likely to have influenced the extent of labeling and the resulting patterns of connectivity. In visual cortex, for example, the posterior location of the injection produced the strongest labeling in LM, LI, and AL, whereas area A was only weakly labeled. The high density of anterograde labeling in AL also increased the likelihood of identifying putative synapses, irrespective of the density of retrograde labeling from M2. Furthermore, the medial-lateral location of the injections also likely influenced the distribution of labeling within extrastriate areas (Supplementary Figures 1, 3, 4; Wang and Burkhalter, 2007; Hovde et al., 2018), and future work could investigate more systematically whether biases in visuomotor connectivity exist for the medial portion of V1, which subtends the peripheral visual field, and lateral V1, which represents the central, binocular field of view. M2-projecting neurons were also labeled based on only one injection site in the posterior sector, chosen due to its known connectivity with visual regions (Reep et al., 1990; Zingg et al., 2014). However, injecting only in posterior M2 may have led to preferential labeling of more medial extrastriate areas and PPC (Olsen et al., 2019), whereas more anterior injections in M2 would likely have labeled more lateral visual areas subtending different parts of the visual field (Leinweber et al., 2017). The injections themselves in V1 and M2 were also densest in intermediate layers (mainly layers 2–5; Supplementary Figures 3, 4), which would favor labeling in matching layers in both up- and down-stream regions (Felleman and Van Essen, 1991; Callaway, 2004). Thus, although we directly observed robust feedforward projections from V1 to extrastriate layers 2/3, projections from V1 to the tips of apical dendrites of layer 5 neurons in layer 1 may have been underrepresented. Because of these constraints, the present study is intended to provide a description of the patterns of labeling in feed-forward visual-to-motor projections, rather than a quantitative account of projection densities or the directionality of connections between visual and motor areas.

Nevertheless, the conjoint use of anterograde and retrograde labeling afforded a direct overview of the anatomical organization of primary visual outputs onto motor cortical inputs in the same preparation. These notably occurred in the same extrastriate regions hypothesized to comprise the dorsal visual processing stream in rodents (Wang et al., 2011, 2012). Intratelencephalic (IT)-to-IT projections, which originate mainly in layers 2/3 and layer 5 (Douglas and Martin, 2004; Harris and Shepherd, 2015), were labeled strongly in our preparations, and the presence of M2-projecting neurons in layers 2/3 and layer 5 suggests that extrastriate and M2 regions participate in mutual feed-forward and feed-back projection pathways (Callaway, 2004; Douglas and Martin, 2004), putting them at nearby stages in the cortical processing hierarchy (Felleman and Van Essen, 1991; Hilgetag et al., 1996). Previous work using dual anterograde tracers showed that regions RL and AM are reciprocally connected with M2, and that all three regions shared directional preferences for visual stimuli and eye movements, suggesting that they comprise an extended sensory-motor network (Itokazu et al., 2018). The frontally projecting neurons we observed in superficial V1 could also participate in mutual feedback loops with frontal motor areas. Based on existing work, such anatomical loops appear to support visual attention (Zhang et al., 2014) and the prediction of expected changes in visual flow due to self-generated movement (Keller et al., 2012; Leinweber et al., 2017).

Although our results provide support for the existence of specialized visual processing streams in rodents, the degree of correspondence with other mammals such as monkeys and carnivores is limited due to considerable differences in brain size, interconnectivity and the relative simplicity of cortical hierarchies in rodents compared to species with more differentiated cortices (Felleman and Van Essen, 1991; Kaas and Krubitzer, 1991; Coogan and Burkhalter, 1993; Krubitzer and Seelke, 2012). For example, whereas visual cortex in primates projects only to nearby higher visual areas and area MT (Felleman and Van Essen, 1991; Hilgetag et al., 2000), V1 in rodents projects to a host of non-visual regions including somatosensory, cingulate, retrosplenial and postrhinal cortices (Vogt and Miller, 1983; Miller and Vogt, 1984; van Groen and Wyss, 1992; Burwell and Amaral, 1998), suggesting a wider and more direct intermixing of visual signals across sensory and cognitive modalities. Thus, visuomotor (Rozzi et al., 2008) and spatial functions (Crowe et al., 2005; Chafee and Crowe, 2012), as well as the reference frames in which they are encoded (Chen et al., 2013), likely follow tighter and more organized anatomical localization in primates and carnivores which, in rodents, appear distributed over coarser topographies. Our present results nevertheless confirm that the anterior and medial extrastriate areas are key sites of visuomotor integration which likely subserve a variety of visually- and spatially guided behaviors in mice.

## Data availability statement

The original contributions presented in this study are included in the article/Supplementary material, further inquiries can be directed to the corresponding author.

## Ethics statement

This animal study was reviewed and approved by the Norwegian Food Safety Authority and the Animal Welfare Committee of the Norwegian University of Science and Technology.

## Author contributions

JW, KH, and MW designed the research. KH and AH performed the research. KH, IR, and AH analyzed the data. JW, KH, and IR wrote the manuscript with input from MW and AH. All authors contributed to the article and approved the submitted version.
