# Supplementary material for: Visuomotor interactions in the mouse forebrain mediated by extrastriate cortico-cortical pathways
Source: Front Neuroanat. 2023 May 9;17:1188808. doi: 10.3389/fnana.2023.1188808 (PMC10203190; doi:10.3389/fnana.2023.1188808)
Supplement: Supplementary file 1 [file Data_Sheet_1.pdf]

Supplementary Material

Visuomotor interactions in the mouse forebrain mediated by extrastriate cortico-cortical pathways

Karoline Hovde[1,2], Ida V. Rautio[1], Andrea M. Hegstad[1], Menno P. Witter[1], *Jonathan R. Whitlock[1]

* **Correspondence:** Jonathan R. Whitlock: jonathan.whitlock@ntnu.no

Supplementary Figures 1-7

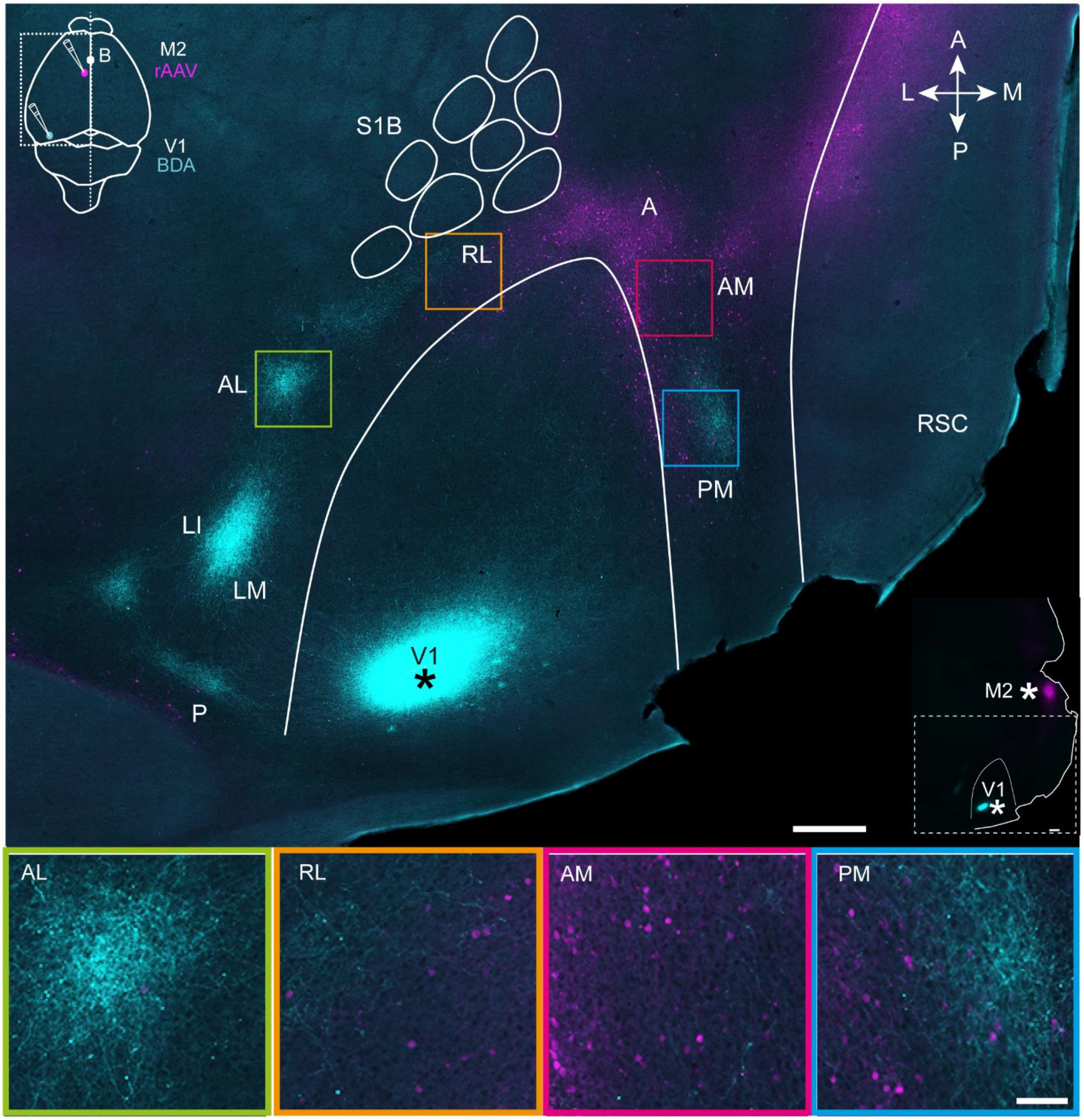

Supplementary Figure 1. Projections from V1 (cyan) and M2-projecting neurons (magenta) viewed in a tangential section of flattened dorsal cortex of same brain as Figure 1 (mouse #83487). Top, a tangential section through layer 4 of flattened cortex shows the BDA injection site in V1 (marked with asterisk; see also inset at top left) and projections to extrastriate areas at the periphery of V1. M2-projecting neurons are shown in magenta (see injection schematic in inset), and colocalized with V1 efferent fibers in areas AL, RL, AM and PM. The outlines of V1 and the S1 barrel fields were traced using myeloarchitectonic patterns and M2AChR staining (Methods) and copied over to the neighboring section in Figure 1. When scanning the section, shorter exposure time was applied for the injection site than for projections to avoid saturation of signal (Methods). Bottom, magnification of extrastriate areas highlighted in the flattened section above. See list for abbreviations. White scale bar in upper image = 500 µm; in lower image = 100 µm.

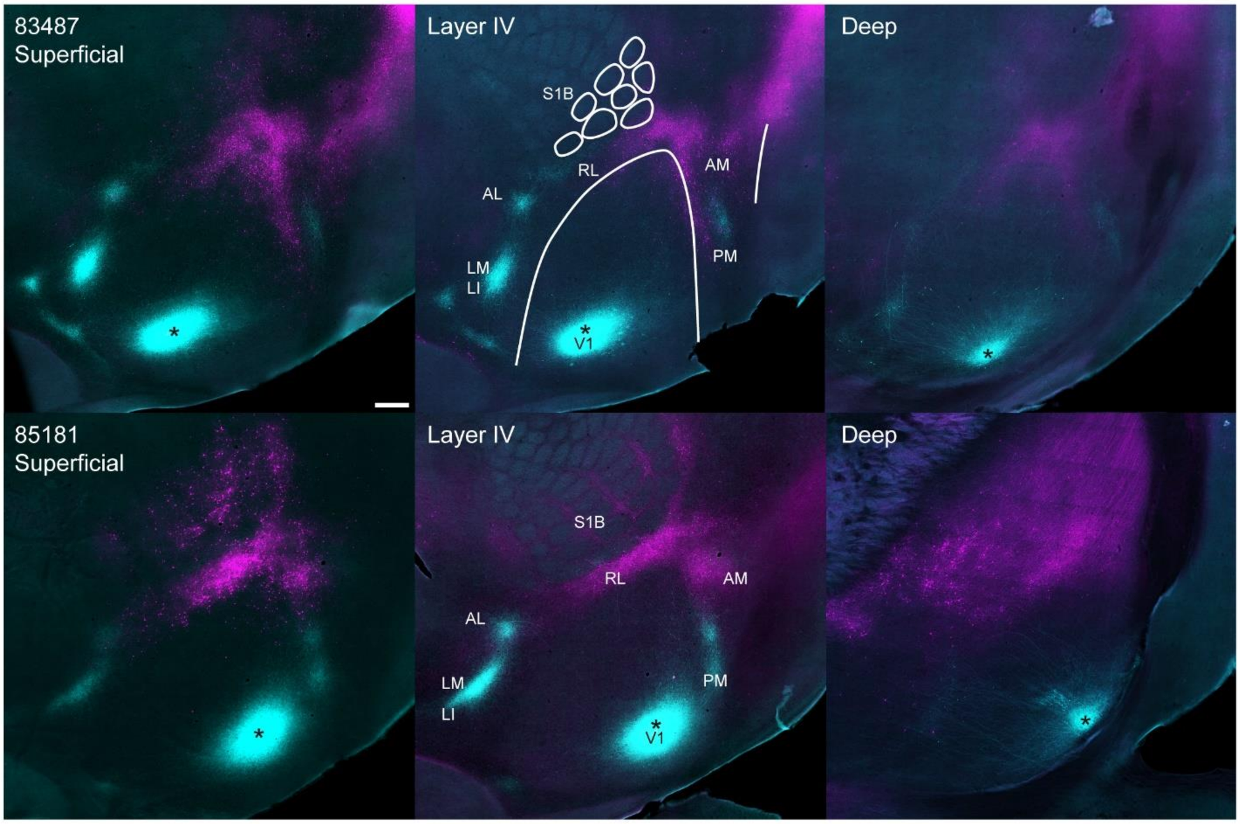

Supplementary Figure 2. Projections from V1 (cyan) and M2-projecting neurons (magenta) viewed in three dorsoventral levels of tangential sections through flattened dorsal cortex. Top (left to right), superficial layers, layer 4 and deep layers of the same brain from Figure 1 (mouse #83487). Outlines of V1 and S1B were drawn using myeloarchitectonic patterns from layer 4 (top middle); the injection site in V1 is marked with asterisk. Bottom (left to right), superficial layers, layer 4 and deep layers from a second mouse brain (#85181) with a more medial injection site in V1, indicated with asterisk. See list for abbreviations. Scale bar = 500 µm.

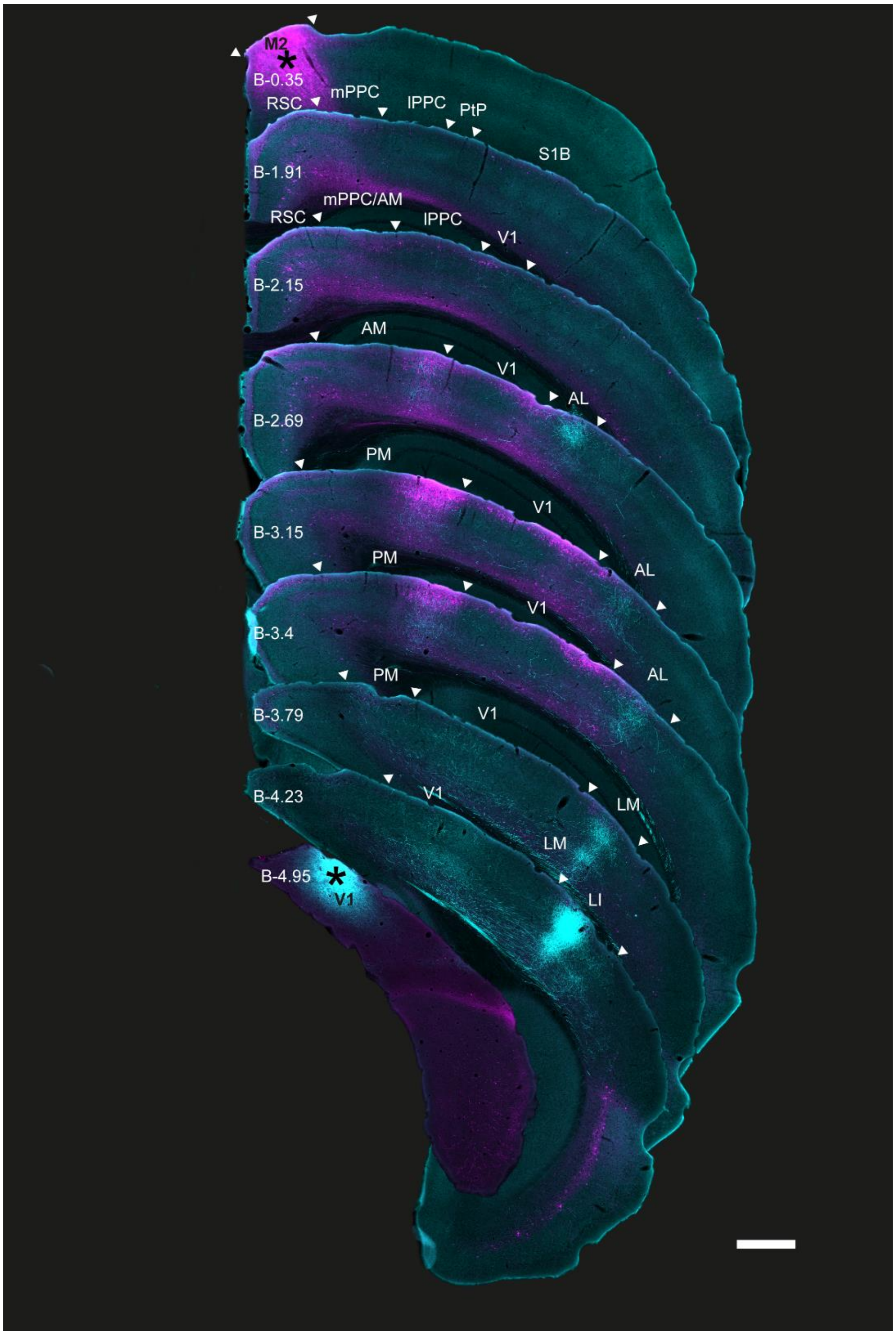

Supplementary Figure 3. Coronal sections showing the laminar profile of BDA-labeled V1 projections and rAAV-retro-labeled M2-projecting neurons in posterior cortices (mouse #81236). A low magnification series of coronal sections arranged from anterior (top) to posterior (bottom) from the right hemisphere; injection sites in M2 and V1 are marked with asterisks. Extrastriate and PPC boundaries are indicated by white triangles; PPC, its sub-areas, and V1 were delineated using adjacent Nissl and immunohistochemically stained sections from the same series (Methods). As with Figure 1, shorter exposure times were used for injection sites than for projections to avoid signal saturation (Methods); the figure is for illustration purposes. See list for abbreviations. Approximate Bregma coordinates (per Paxinos and Franklin, 2012) are noted on each section; scale bar = 500µm.

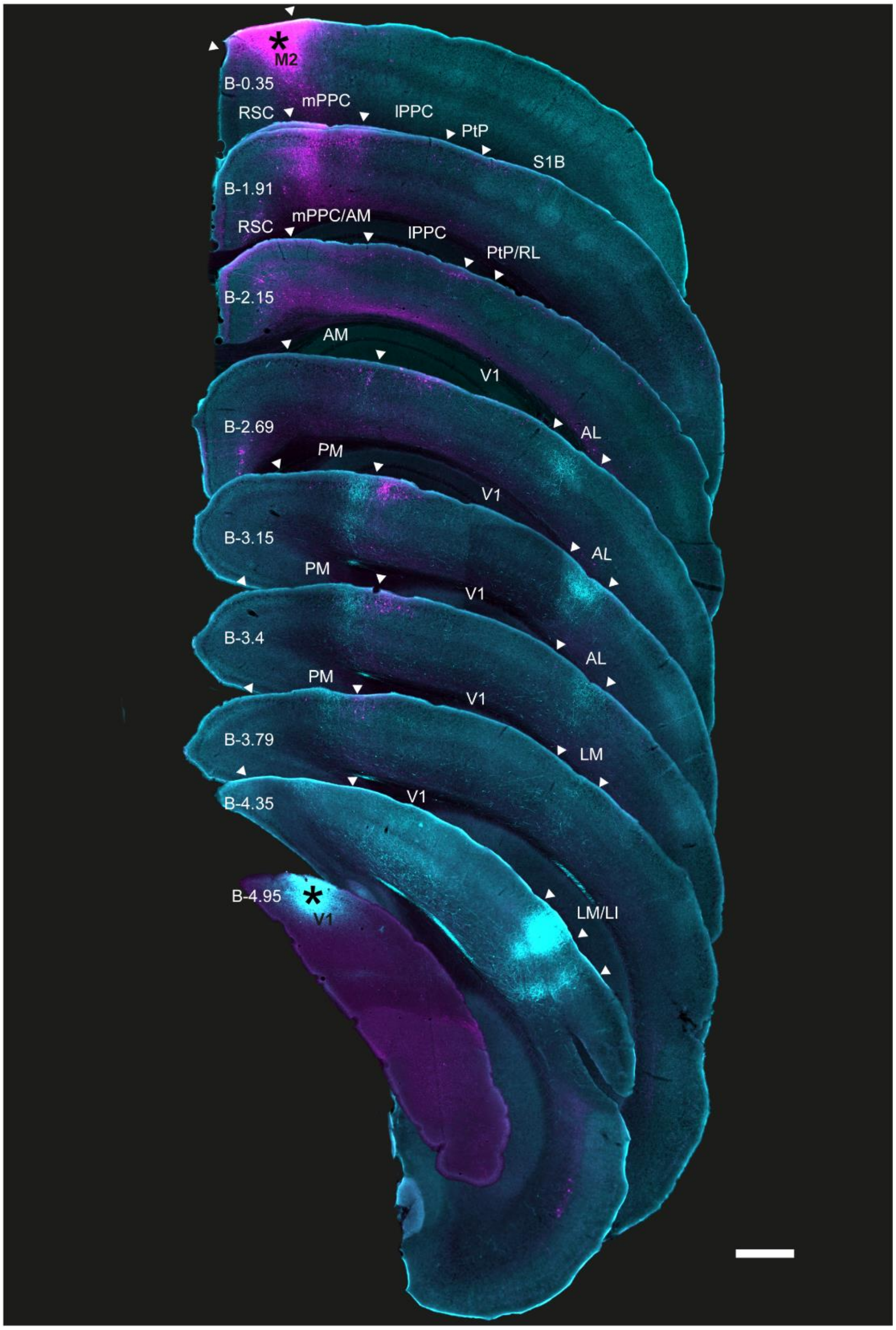

Supplementary Figure 4. **Coronal sections showing the laminar profile of BDA-labeled V1 projections and rAAV-retro-labeled M2-projecting neurons in posterior cortices (mouse #81233).** Low magnification series of coronal sections arranged from anterior (top) to posterior (bottom) from the right hemisphere; injection sites marked with asterisks. Extrastriate and PPC boundaries are indicated by white triangles; PPC, its sub-areas, and V1 were delineated using adjacent Nissl and immunohistochemically stained sections from the same series (Methods). As with Figure 1 and Supplementary Figure 3, shorter exposure times were used for injection sites than for projections to avoid signal saturation (Methods); the figure is for illustration purposes. See list for abbreviations. Approximate Bregma coordinates (Paxinos and Franklin, 2012) are noted on each section; scale bar = 500µm.

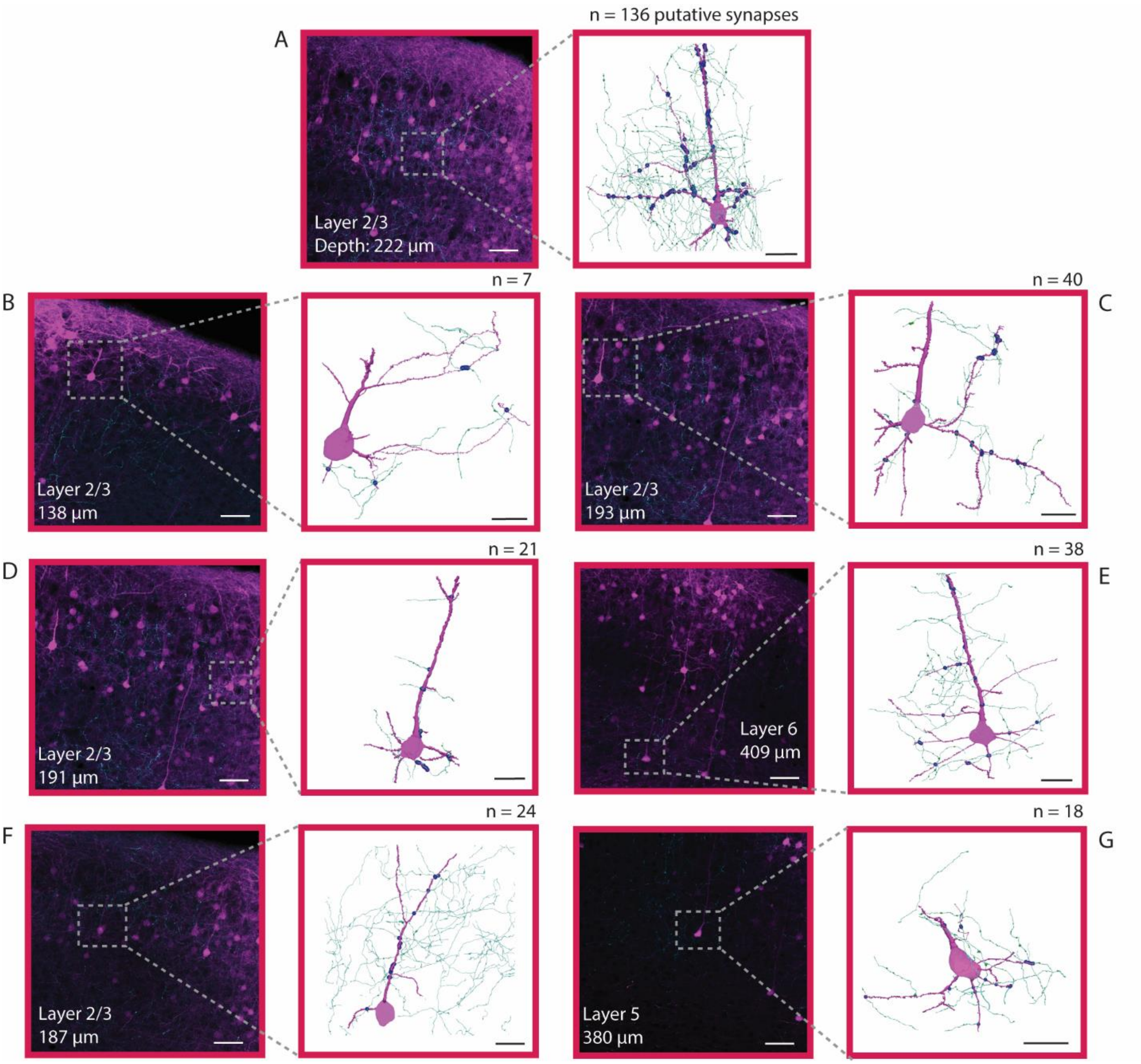

Supplementary Figure 5. Anatomical 3D reconstructions of V1 axons and seven M2-projecting neurons in extra striate area AM. Left, inset, 20x magnified field of view highlighting the portion of area AM where each cell was identified for reconstruction. Right, reconstructions of single M2-projecting pyramidal neurons from area AM (magenta) receiving synaptic input from V1 axons (cyan); putative synaptic contacts are shown as blue circles. Number of putative synapses (markers with a distance below 0.25µm) noted above each reconstruction. The depth (from cortical surface to soma center) and cortical layer of each neuron are included in the inset for each example. A-D: mouse #81234, E: mouse #81236, F-G: mouse #81233. Cells from deep layers: E and G. White scale bars = 50 µm. Black scale bars = 20 µm.

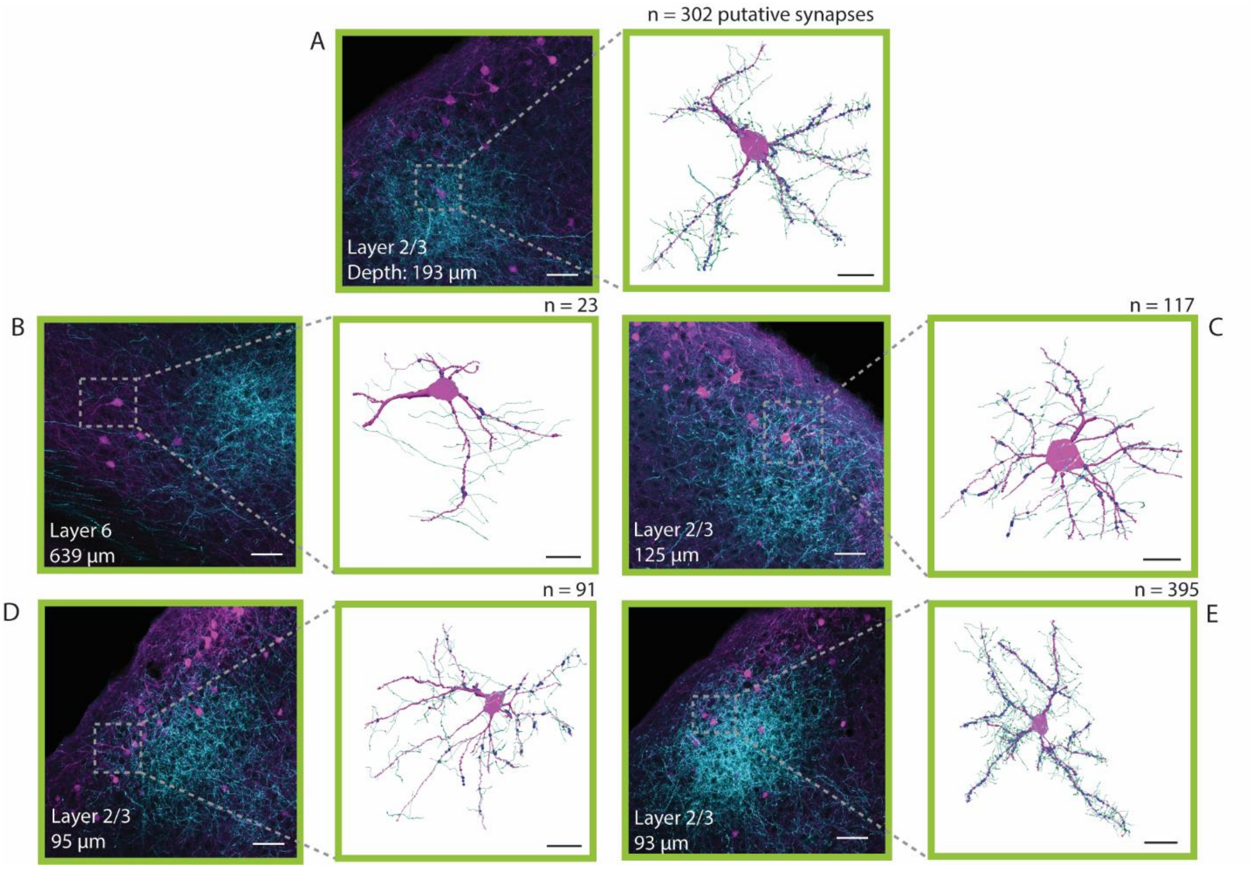

Supplementary Figure 6. Five anatomical 3D reconstructions of V1 axons and M2-projecting neurons in extrastriate area AL. Left, inset, 20x magnification field of view highlighting the portion of area AL where the neurons were identified for reconstruction. Right, reconstructions of single M2-projecting pyramidal neurons from area AL (magenta) receiving synaptic input from V1 axons (cyan). Putative synaptic contacts are indicated by blue circles. The number of putative synapses (i.e. axon and dendrite segments separated by < 0.25µm) is noted above each reconstruction. The depth (from cortical surface to soma center) and cortical layer of each neuron are included in the inset for each example. Note that the neuron in panel C is the same example neuron as in Figure 3A. A-C: mouse #81234, D-E: mouse #81236. B: deep layer neuron. White scale bars = 50 µm. Black scale bars = 20 µm.

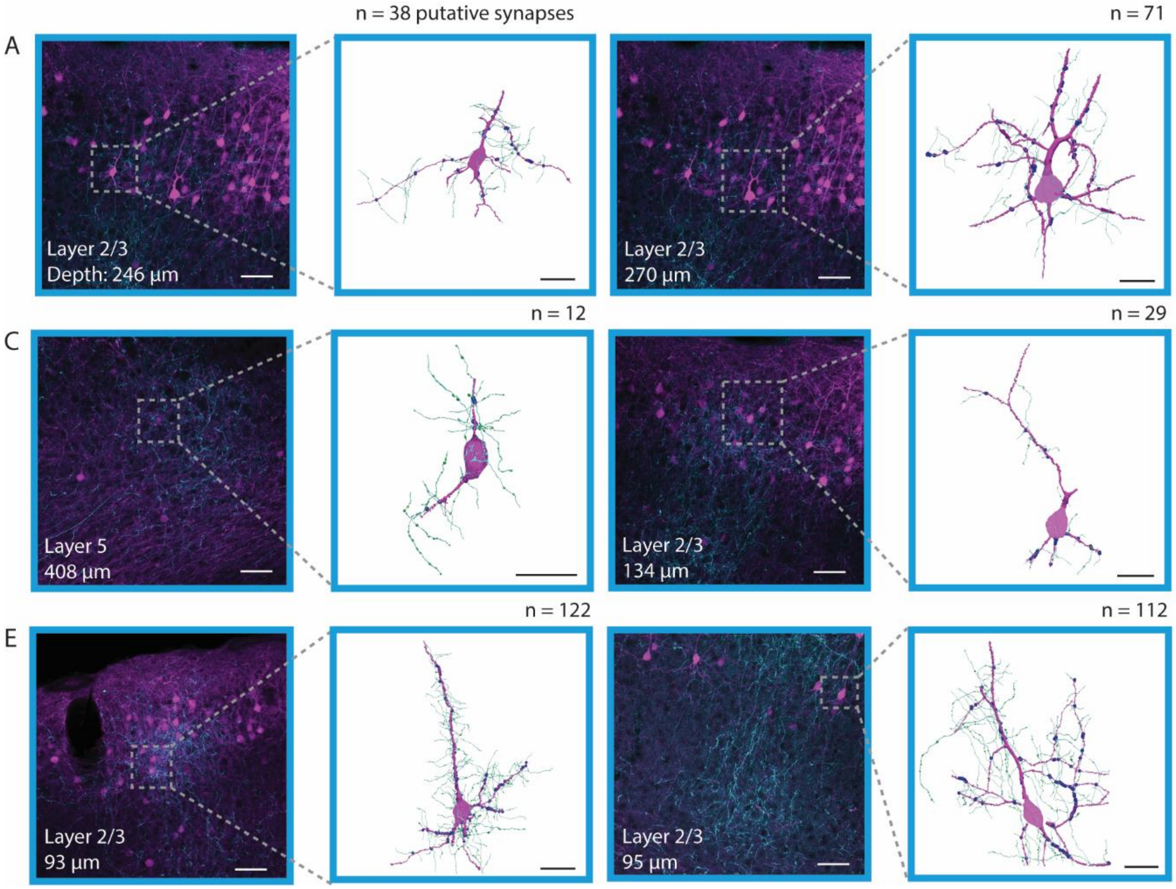

Supplementary Figure 7. Six anatomical 3D reconstructions of M2-projecting neurons axons from V1 in extrastriate area PM. Left, inset, 20x magnified field of view highlighting the portion of area PM where each cell was identified for reconstruction. Right, reconstructions of single M2-projecting pyramidal neurons in area PM (magenta) receiving synaptic input from V1 axons (cyan); putative synaptic contacts are shown as blue circles. The number of putative synapses (markers with a distance < 0.25µm) is noted above each reconstruction. The depth (from cortical surface to soma center) and cortical layer of each neuron are included in the inset for each example. A-D: mouse #81234, E: mouse #81236, F: mouse #81233. Deep layer neurons: C. White scale bars = 50 µm. Black scale bars = 20 µm.
